# Game-based cognitive training for the aging brain

**DOI:** 10.3389/fpsyg.2014.01100

**Published:** 2014-09-29

**Authors:** Julia Karbach

**Affiliations:** Department of Educational Science, Saarland UniversitySaarbruecken, Germany

**Keywords:** cognitive training, cognitive plasticity, cognitive aging, game-based learning, executive functions

Cognitive aging is associated with a decline in cognitive control functions (Daniels et al., 2006), including working memory, inhibition, and cognitive flexibility (Miyake et al., 2000). Given that impairments in cognitive control are associated with impaired functioning in daily life (Burgess et al., 1998), numerous cognitive training studies aimed at improving cognitive control in older individuals. They showed that cognitive plasticity is considerable up to very old age and that cognitive control training leads to significant performance improvements on the trained tasks. Moreover, some interventions benefitted performance on untrained tasks that were tapping the same ability as the training tasks (near transfer) or even other abilities (far transfer) (see the Frontiers Research Topic on training-induced cognitive and neural plasticity, Karbach and Schubert, 2013). However, transfer effects were not consistent across studies and have inspired heated recent debates (e.g., Shipstead et al., 2012; Redick et al., 2013).

These inconsistent findings of previous studies may be explained by large differences in the type and the duration of their training regimes. For instance, strategy-based memory trainings (e.g., by means of mnemonic techniques) often resulted in large, long-lasting training gains but rarely in any transfer (Verhaeghen et al., 1992; Rebok et al., 2007). Process-based trainings, targeting more general processing capacities, such as cognitive flexibility or working memory, have yielded widespread transfer in different age groups (see Hertzog et al., 2009; Karbach and Unger, 2014). One increasingly popular type of training is multi-domain training, engaging multiple cognitive processes and including most game-based trainings. Even though the complexity of multi-domain trainings makes it hard to determine the specific features inducing the training-related gains, they are considered an optimal context for cognitive interventions because of their stimulus variability, frequent feedback and motivating nature. Positive effects of game-based training have been repeatedly reported for younger and older adults (Green and Bavelier, 2003; Basak et al., 2008; Strobach et al., 2012; Anguera et al., 2013; for a review see Kueider et al., 2012).

To improve cognitive control in older adults, van Muijden et al. (2012) developed a training regime including five online training games designed to tap updating, shifting, and working-memory maintenance. The games were adaptive in the sense that the task difficulty was individually adapted to participants' performances to make sure that they were constantly challenged and performing close to their individual limit without being overstrained. A sample of more than 70 older adults (mean age ≈68 years) either performed 30 min of game training per day for 7 weeks (training group), or they spend the same amount of time watching documentaries and answering quiz questions online (active control group). Before and after the training, participants completed a battery of cognitive tests assessing working memory, inhibition, shifting, visual attention, and fluid intelligence.

After the intervention, the training group improved more than the control group in terms of fluid intelligence and response inhibition, indicating that the training resulted in far transfer to these abilities. It should be noted, however, that the inhibitory benefits were associated with significant baseline differences between the training and the control group that disappeared after the training. Interestingly, the control group improved more in terms of selective attention, which the authors attributed to the participants in the control condition being required to constantly and simultaneously monitor multiple stimuli.

Thus, the study provided limited support for positive effects of game-based training on cognitive control in older participants. It is consistent with Basak et al.'s (2008) findings that game playing can improve inhibition and other previous data suggesting that fluid intelligence can be improved by process-based cognitive training (e.g., Klingberg et al., 2005; Jaeggi et al., 2008; Karbach and Kray, 2009; Schmiedek et al., 2010). However, van Muijden's findings are inconsistent with previous research claiming that game-based training does not benefit cognitive functioning (e.g., Ackerman et al., 2010; Owen et al., 2010). These differences may be related to methodological flaws that particularly characterized Owen's study, among them large differences in terms of compliance and motivation in the study sample. This issue is of particular importance because recent work has identified motivation as a key condition for transfer to occur (Green and Bavelier, 2008; Jaeggi et al., 2014). In contrast, most of the participants in van Muijden's methodologically sound study reported enjoying the training games, a fact that may have supported the occurrence of transfer.

One matter that may have explained the finding of limited transfer in van Muijden's study is an analysis of individual differences in training and transfer gains. Like most previous studies, the authors focused on between-group differences in training-related gains without analyzing for whom the training was most effective. Process-based training often yields compensatory effects with larger benefits in low-performing participants (see Titz and Karbach, 2014). Also, recent work indicated that transfer is more likely to occur in participants with large gains on the training tasks (Jaeggi et al., 2011). Hence, some individuals may have shown large transfer effects that were masked in the analyses based on group means. Identifying these individuals may provide important information for the understanding of the mechanisms mediating transfer to untrained tasks.

Even though recent studies indicated that game-based training may be a promising tool to enhance cognitive abilities in older adults, transfer effects were not consistent and in some cases rather limited (Kueider et al., 2012). Thus, should we consider game-based training ineffective in older adults? Based on what we know so far, the answer to this question clearly should be no. The existing research still is too divers to come to any kind of strong conclusion regarding the potential of game-based interventions in older age. Instead, we need to further explore which specific features of a game yield training-induced gains, how long these effects last, who benefits the most and how interventions have to be designed to benefit groups at risk (e.g., individuals suffering from mild cognitive impairment or neuropsychiatric disorders). Many of these questions will be addressed in an upcoming Frontiers Research Topic on effects of game training on neurocognitive plasticity (Band et al., in press).

## Conflict of interest statement

The author declares that the research was conducted in the absence of any commercial or financial relationships that could be construed as a potential conflict of interest.
